# Convolutional Neural Network-Based Artificial Intelligence for Classification of Protein Localization Patterns

**DOI:** 10.3390/biom11020264

**Published:** 2021-02-11

**Authors:** Kaisa Liimatainen, Riku Huttunen, Leena Latonen, Pekka Ruusuvuori

**Affiliations:** 1 Faculty of Medicine and Health Technology, Tampere University, FI-33520 Tampere, Finland; kaisa.liimatainen@tuni.fi (K.L.); riku.huttunen@uef.fi (R.H.); 2 Department of Applied Physics, University of Eastern Finland, FI-70211 Kuopio, Finland; 3 Institute of Biomedicine, University of Eastern Finland, FI-70211 Kuopio, Finland; leena.latonen@uef.fi; 4 Institute of Biomedicine, University of Turku, FI-20014 Turku, Finland

**Keywords:** protein localization, artificial intelligence, convolutional neural networks, deep learning, phenotyping, classification, fluorescence microscopy, cellular organelles

## Abstract

Identifying localization of proteins and their specific subpopulations associated with certain cellular compartments is crucial for understanding protein function and interactions with other macromolecules. Fluorescence microscopy is a powerful method to assess protein localizations, with increasing demand of automated high throughput analysis methods to supplement the technical advancements in high throughput imaging. Here, we study the applicability of deep neural network-based artificial intelligence in classification of protein localization in 13 cellular subcompartments. We use deep learning-based on convolutional neural network and fully convolutional network with similar architectures for the classification task, aiming at achieving accurate classification, but importantly, also comparison of the networks. Our results show that both types of convolutional neural networks perform well in protein localization classification tasks for major cellular organelles. Yet, in this study, the fully convolutional network outperforms the convolutional neural network in classification of images with multiple simultaneous protein localizations. We find that the fully convolutional network, using output visualizing the identified localizations, is a very useful tool for systematic protein localization assessment.

## 1. Introduction

A major determinant of protein functions is their localization in and traffic between intracellular compartments specialized in certain cellular tasks. Thus, identifying protein localizations and specific subpopulations associated with certain cellular compartments is often a key task in protein research.

Fluorescence-based imaging techniques are the norm in protein localization studies due to their superior ability to visualize intracellular proteins, either through the expression of fluorescent fusion proteins or recognition of target proteins by fluorophore-detected, antibody-based techniques. Considering that the human genome contains approximately 20,000 protein-coding genes, that many of these proteins can be expressed as several protein isoforms, and that each protein can, in principle, be recognized by several antibodies identifying specific and possibly different functional forms (e.g., antibodies specific for certain conformation or post-translationally modified forms), the number of protein populations to study in localization assays is enormous.

Recently, systematic and high throughput approaches to visualize all human proteins in cells have surfaced [1]. The visualization and systemic analysis of such vast numbers of protein localizations already require better, high throughput tools. Despite the merit of the current studies, more data is required for assessing cell type and state-specific protein localizations, and variation during e.g., disease states. It is expected that future analyses will further increase the amount of protein localization image data significantly. Thus, ways to automatically classify protein localization in fluorescence microscopy images with high throughput analysis methods are required.

Phenotype analysis of high content screening images poses a challenge for image-based classification systems. The traditional approach of quantifying the images on a single cell level by representing the cells as a set of features and using the feature representation for phenotype analysis has been successfully applied in several studies, e.g., in [2,3,4]. Tools such as CellProfiler [5,6], CellClassifier [7], CellCognition [8] and Advandced Cell Classifier [9] along with other open-source and commercial projects have enabled the use of machine learning-based phenotype characterization. Extending and tailoring the tools for specific tasks is possible but, as with any complex classification tasks, becomes very challenging with multiple classes and high sample heterogeneity. Lately, the traditional approach has been superseded by deep learning (DL)-based artificial intelligence (AI) methods [10] in many application areas in biomedicine (see e.g., [11,12,13,14,15,16,17] for a variety of examples), including image-based detection and classification tasks [18,19,20]. The success of deep learning-based methods has been evidenced in several large public contests, which basically offer a crowd-sourcing-based approach for finding effective computational methods for specific tasks [21,22]. The overwhelming success of deep learning has made the traditional feature-based machine learning approach almost redundant in data-rich applications.

Convolutional neural networks (CNN) have emerged as most common and successful tool for image classification tasks in recent years, especially in data-rich applications [21,23]. Usually, CNNs are used to predict which class is present in an image. By discarding fully connected layers of CNN we can create a fully convolutional network (FCN) which outputs a predicted heat map, showing also the spatial locations where detected classes are present. FCNs have been successful especially in biomedical image analysis [19,24], and they have also been experimented with in the field of object detection [25]. Usually, the type of classification problem defines which approach, CNN or FCN, is better for the task. The defining factor is the ground truth data: if only image-wise ground truth is available, binary targets for FCNs cannot usually be created. If, on the other hand, pixel-wise ground truth exists, either CNNs for pixel-wise predictions can be used, a common approach in cancer detection from stained tissue sections [21,26,27], or the binary mask can be used as a training target for FCN. While this work focuses on comparing standard CNN and FCN architectures, architectures like Resnet, Densenet and Inception would also be suitable for this type of classification task [28,29,30]. For example, the best performing solutions in Human Protein Atlas Image Classification competition [22] were based on these three architectures.

Here, we studied the prediction of protein signal localization for the Cell Atlas data from Human Protein Atlas (www.proteinatlas.org accessed on 10 February 2021) [1,31] using two standard deep neural network-based AI-approaches (CNN, FCN) in a comparative manner. With Cell Atlas data, training targets can automatically be created for FCNs from a fluorescence channel. Thus, even though only image-level class information is available, the spatial target information needed for FCN can be generated, enabling the use of both CNN and FCN for the classification task. In addition to the very interesting biological aspects of the Cell Atlas data [1], it enables comparison between FCNs and CNNs in the classification of localization in fluorescence images of thousands of proteins. We found both network architectures performing equally well for the identification of protein localization in most cellular structures. Furthermore, we identified challenges for future research in automated localization detection from fluorescent microscopic images.

## 2. Materials and Methods

### 2.1. Data

Data consist of 20,000 samples of human proteins immunostained in cell culture samples to acquire the protein localization patterns for Cell Atlas of Human Protein Atlas. The dataset was provided for Image Analysis Challenge of Cyto conference in 2017. Images were acquired with confocal fluorescence microscopy. Each sample is a four-channel confocal image. Image with green color shows the localization pattern of the protein of interest, labeled with immunofluorescence. Other channels are provided for reference: channel represented in blue color showing nucleus counterstained with DAPI, channel in red with microtubules marked with antibody against tubulin, and channel in yellow showing endoplasmic reticulum (ER) [1].

**Figure 1:**
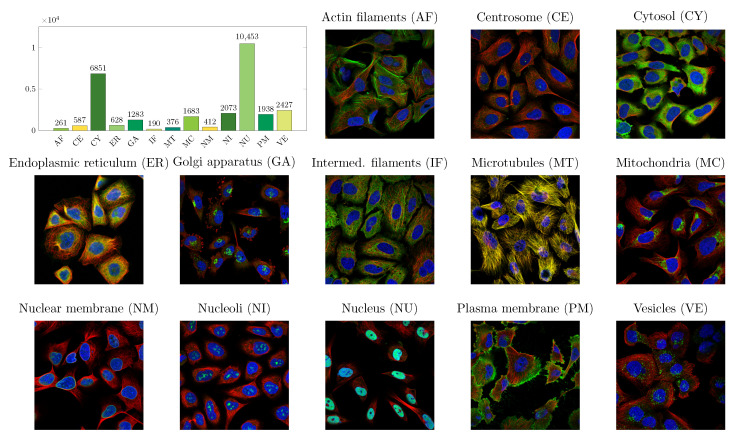
Cell organelle distributions (graph) in the dataset reveals imbalance between phenotype classes. Color coding is for visual separation of the bars. Example images of each cell organelle, written in image titles, show characteristic localization pattern of each phenotype. Blue color channel shows the nucleus and red channel presents microtubules. Green channel presents the protein of interest, labeled with immunofluorescence. Yellow reference color channel showing endoplastic reticulum was not used in images, however, green and red channels in same locations result in yellow color. This is especially clear in microtubules image, where both red and green colors mark microtubules.

In this dataset, protein distributions were classified into 13 major organelles of the cell: actin filaments (AF), centrosome (CE), cytosol (CY), endoplasmic reticulum, golgi apparatus (GA), intermediate filaments (IF), microtubules (MT), mitochondria (MC), nuclear membrane (NM), nucleoli (NI), nucleus (NU), plasma membrane (PM), and vesicles (VE). The protein localization classes with examples of each class (RGB images showing also the reference stainings for microtubules, fluorescence signal, and nucleus), and distribution of classes are presented in Figure 1. Data was annotated by EVE online gamers within a mini-game giving in-game prices for motivation as explained in [31]. Briefly, the gamers participating in the mini-game were trained via examples and true classes were decided via majority vote of gamers.

**Figure 2:**
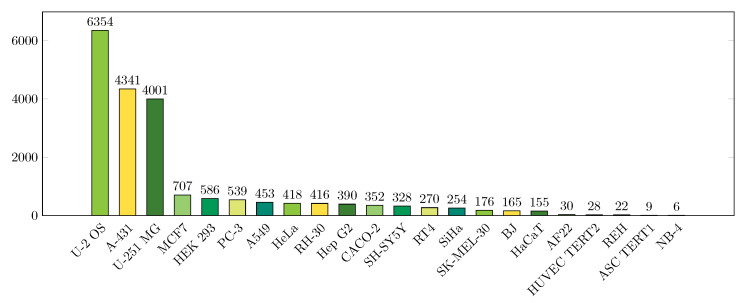
Cell line distribution shows that majority of the images in the dataset originate from three cell lines. Color coding is for visual separation of the bars.

In total, 22 different cell lines were used to create the dataset. Majority of the samples belong to either U2-OS, A-431, or U-251 MG cell lines. The distribution of samples with respect to the cell lines is presented in Figure 2. Approximately 60 percent of the samples have only a single protein localization class present, and the remaining 40 percent have two to four classes.

### 2.2. Deep Learning

Two different deep learning architectures were used. The base architecture for both networks is a straightforward convolutional neural network with sets of two convolutional layers followed by batch normalization and max pooling layers. The amount of kernels doubles after each max pooling layer. In total, 10 convolutional layers were used in the base network. The depth of the network was chosen experimentally by testing various depths between 4 to 16 convolutional layers. Since one aim of this study was to compare CNN and FCN, a simple architecture that can easily be transformed to closely matching CNN and FCN implementations was chosen. CNN performance could improve with a deeper model, but the FCN output would then be too small to find locations of the cells where a class is present. On the other hand, U-Net [19], for example, is a powerful FCN, but due to upsampling layers the architecture is very different from most powerful CNNs.

**Figure 3:**
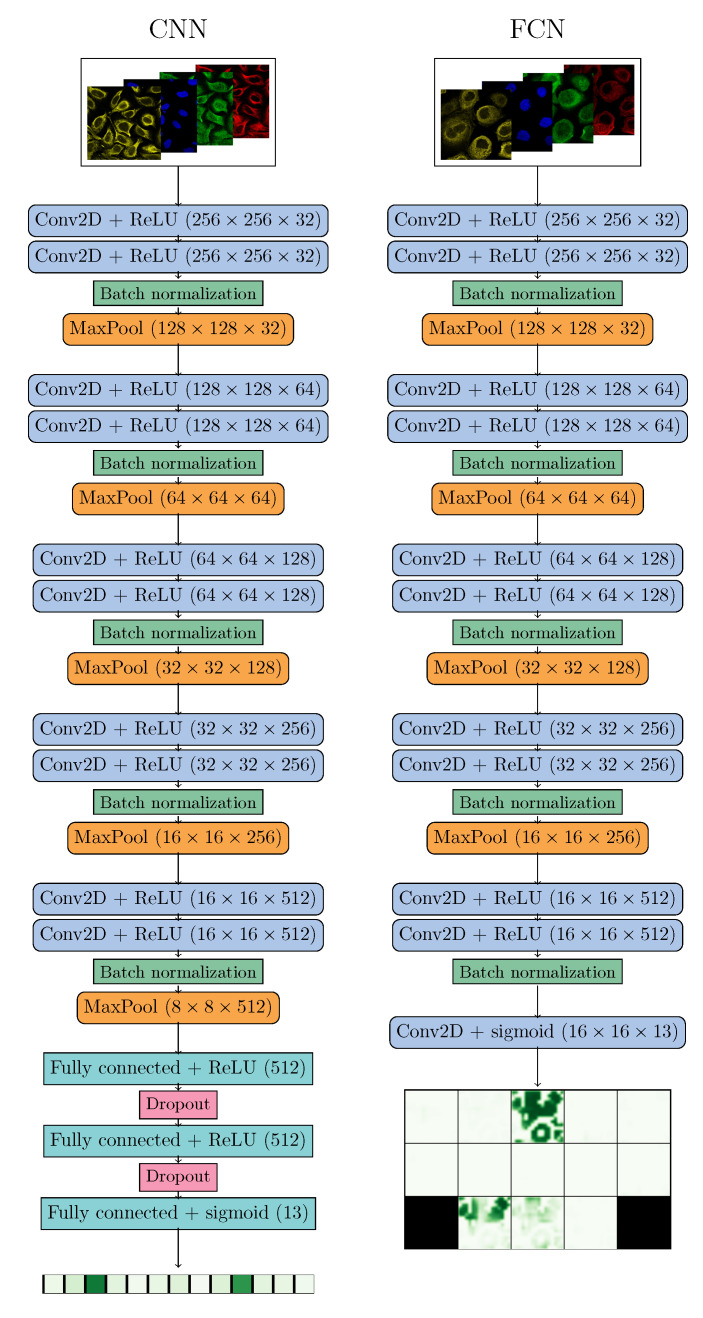
Convolutional neural networks (CNN) (**left**) and fully convolutional network (FCN) (**right**) architectures. An example output for a real input image is presented for FCN.

From the base architecture, CNN was created by adding three fully connected layers to the end of the network, last being the predicting layer with 13 output values. FCN was created by removing the last max pooling layer to increase the output size, and adding an additional convolutional layer with 13 kernels to the end of the network, one for each class. Both architectures are illustrated in Figure 3. Rectified linear unit (ReLU) activation was used after each convolutional layer, except for the final layers where sigmoid activation was performed to map the output between zero and one.

All four channels were used as input for the networks, and were resized to 256×256 pixels. Original image sizes were 1728×1728, 2048×2048 and 3072×3072 pixels, and were resized with nearest neighbour resampling. Tiling strategies could not be used since proteins might not locate in same compartments of the cells in one image and, thus, a single tile might not include all of the classes present. CNN outputs a single confidence value for each of the 13 classes, while FCN outputs 13 heat maps of size 16×16 pixels. The binary training targets for FCN were acquired from fluorescence channels with basic image processing methods. First, the image was normalized and filtered with Gaussian kernel. Then, the smoothened image was thresholded with experimentally selected value 0.3. Finally, the binary image was dilated and resized to match the output size. The same target was used for each class present, while classes not present were given zeros as target.

The codes implementing the models used in this study are available at https://gitlab.com/BioimageInformaticsGroup/cytoclassifier/ (accessed on 10 February 2021).

### 2.3. Training

In addition to the network architecture, also the details of training process are key factors in understanding the implementation and in determining the performance. Here, we present the parameter choices used when training the deep neural networks.

From the 20,000 sample data set we used 16,000 samples for training the models. From these samples, 10 percent were used for validation during training. The remaining 4000 samples were used for testing only. The data was divided into train and test sets simply by indices of the images, using first 16,000 images for training. The same division to training and test set was used for both models. The class distribution in train and test sets is very similar to overall class distribution shown in Figure 1.

The networks were trained from scratch, without using transfer learning from pre-trained models. However, we also experimented with transfer learning approach using Inception V3 network [30] that was pre-trained with ImageNet data without a gain in the performance.

The hyperparameters for training were tuned experimentally. The training process was run for 30 epochs and the hyperparameters providing the lowest validation loss were used for final training. First, an initial hyperparameter combination was chosen, with the principle of limiting the network complexity. Then, a suitable number of layers was searched using Adam optimizer with default parameters. Next, a trade-off between the number of kernels (doubling after each max pooling for memory reasons) and the batch size was searched. After that, batch normalization layers were added. Then, 5 × 5 filter size was tested against the initial 3 × 3 filters. Activation function was chosen by comparing leaky ReLU and exponential linear unit (ELU) with the default ReLU, and the optimizer was chosen by comparing SGD and Adam optimizer. The dropout parameter value was optimized in a grid search (0.1, 0.2, 0.3, 0.5). Finally, different learning rate decay factors, as well as the number of epochs without improvements to wait before decaying the learning rate were experimentally defined. The final model was trained for 100 epochs with the chosen hyperparameter combination. The combination of the structural parameters and the batch size is a compromise dictated by the GPU memory available, since the whole batch needs to be handled by the GPU in a single go for performance reasons.

The final choices used for model training were the following. The model weights were initialized with Glorot initialization [32]. The models were trained using Adam optimizer [33]. The default parameters suggested by the original Adam paper (learning rate = 0.001, beta1 = 0.9, beta2 = 0.999, epsilon = $1.0\times 10^{-8}$ were used initially [33]. The learning rate was divided by three when the validation loss did not decrease for 8 consecutive epochs. Binary crossentropy was chosen as loss function due to multi-label nature of the data. Binary crossentropy is also a well-suited loss function for FCNs with binary targets. Batch normalization with batch size of 64 was used, which was the largest possible batch size for GPU with 12 GB memory. The models were implemented in Python with Keras module using Tensorflow backend [34,35], and GPUs were used for accelerated training. For CNN the confidence values are the direct outputs of the model, while for FCN the confidence is maximum value in predicted heat map of each class. Output values are always between zero and one due to sigmoid activation function after final layer. The confidence thresholds were selected for each class separately in a data-driven manner by testing which value produced highest dice coefficient [36,37] for training data. The interval used for experimenting the threshold values was 0.01.

## 3. Results

We studied the efficiency of using convolutional neural networks for classification of protein localization patterns in a large scale. For this, we utilized a dataset from Cell Atlas composed of 20,000 confocal microscopy images containing information on four channels. There are 13 cellular localizations, or subcompartments, to which each target protein is annotated to reside in. However, the target proteins may have several localizations in a given image.

**Figure 4:**
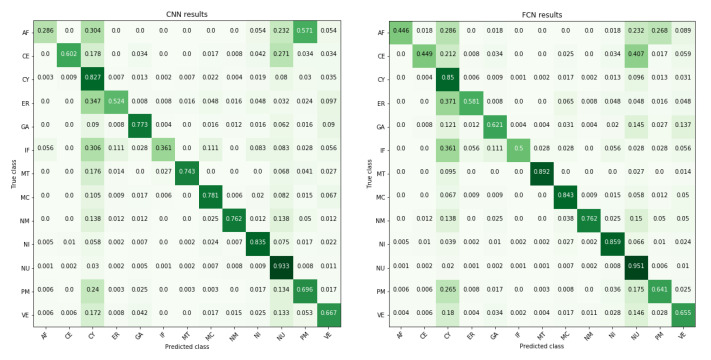
Prediction results as confusion matrices reveal which classes are often mixed with others. In case of multiple labels in sample, each true label not found gets a hit from all predicted false positives. The diagonal shows percentage of correctly classified samples. Mean of diagonal (unweighted class average) is 0.676 for CNN and 0.696 for FCN. Darkness (value) of color represents percentage; higher percentages are depicted with darker color.

To understand what type of convolutional network suits the protein intracellular localization task, we compared CNN and FCN networks. Performance of the CNN and FCN networks in detecting the annotated localizations is shown in Figure 4. Mostly, the performance between the networks is highly similar. Both networks misclassify signals mostly as cytoplasm and nucleus, which are the most common classes in the data (see plot in Figure 1). It is noteworthy that both networks have a high misclassification rate for signals annotated as actin filaments to be classified as plasma membrane. CNN has a slightly higher misclassification rate for this, while FCN has higher misclassification rates than CNN for misclassifying centrosome and Golgi apparatus as nuclear signal. The overall accuracies are 0.676 for CNN and 0.696 for FCN, while unweighted mean of dice coefficients was 0.705 for CNN and 0.707 for FCN. In general, the localization classes with lowest number of cells in training data (such as actin filaments, endoplasmic reticulum, intermed. filaments; refer to Figure 1 for class distribution in data) appear to be less accurately predicted by both networks. It is a well known property that more data typically leads to better models, as is also supported here by the higher prediction accuracies obtained for the larger sample size classes cytosol and nucleus.

**Figure 5:**
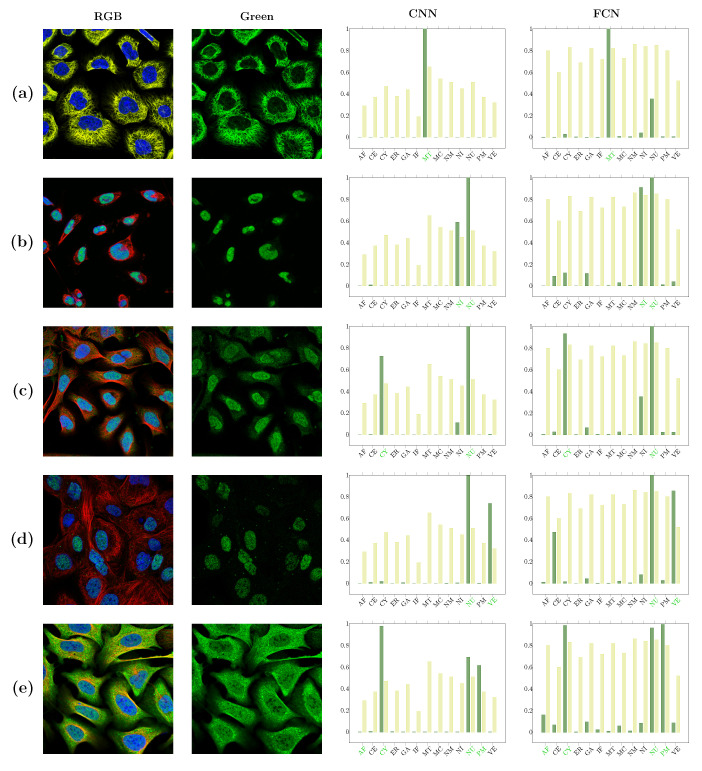
Examples of successful classification. Confidence values are represented with dark green in the plots, and thresholds for each class are lighter bars. The annotated classes are labeled in green. Successful examples include sites with a good reference due to additional stainings, such as microtubules (**a**) and nucleus (**b**–**e**); localization to major classes such as the cytoplasm (**c**,**e**); and clearly distinguishable phenotypes such as vesicles (**d**). Actin filaments is a rare class and, thus, difficult to detect (**e**).

**Figure 6:**
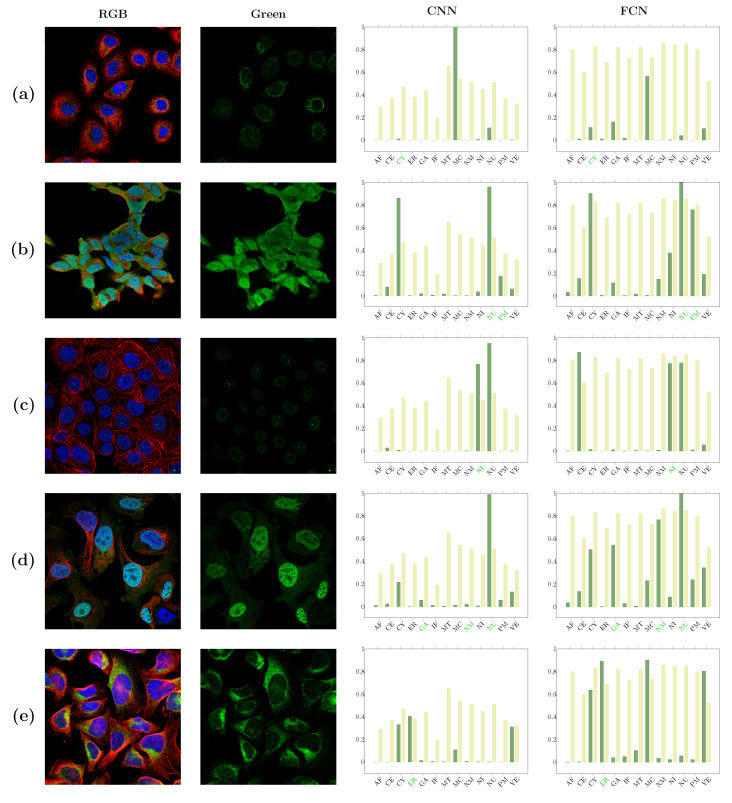
Examples of unsuccessful classification. Confidence values are represented with dark green in the plots, and thresholds for each class are lighter bars. The annotated classes are labeled in green. Unsuccessful classification can result from clear misannotations of the only or one of the several localizations (**a**,**b**) or low signal intensity (**c**,**d**). In (**e**), a “true” misclassification is presented.

In Figure 5, examples of successful classification are shown. These include images with localizations to major classes such as the cytoplasm, clearly distinguishable phenotypes such as vesicles, as well as sites with a good reference due to the additional stainings, such as nucleus and microtubuli. On the other hand, the less successful classification examples include clear examples of misannotations of the only or one of several localizations (Figure 6a,b), misannotation and/or misclassification due to low signal intensity (Figure 6c,d), and what seem as “true” misclassification (Figure 6e). In Figure 5 and Figure 6 the thresholds for each class are represented with lighter bars. Thresholds for CNN are overall much smaller than thresholds for FCN. Some classes are predicted by both networks with confidence of 1, but otherwise the correct predictions of CNN have often smaller confidence, which translates directly to thresholds.

**Figure 7:**
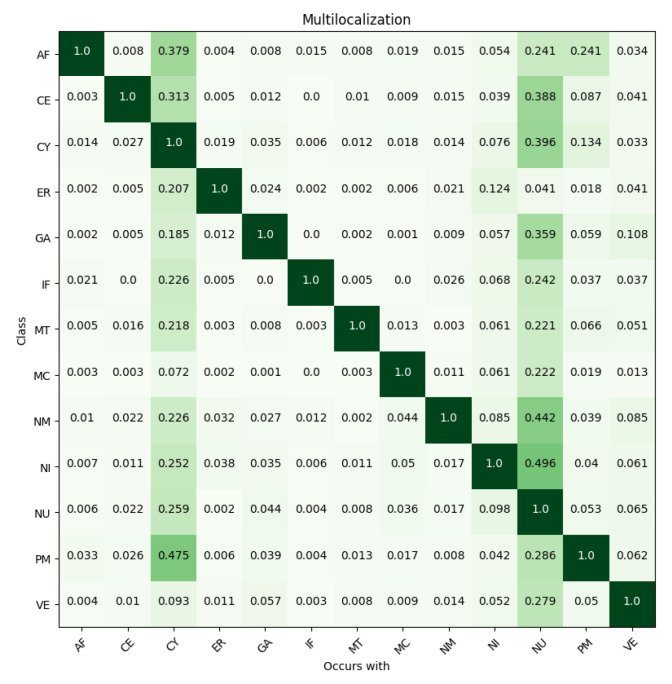
Multilocalization of classes. Rows represent class fractions localizing with classes in columns. Darkness (value) of color represents fraction value; higher fractions are depicted with darker color.

Another biologically interesting, and computationally challenging, property in the data is the multilocalization of proteins. Proteins can localize in multiple subcellular compartments, creating a multi-label classification problem. Multilocalization of proteins is presented in Figure 7, where the fraction of each cell organelle class localizing with other classes is shown. The performance of the classification for both networks was clearly best with images containing only one annotated localization, while the accuracy decreased when the number of simultaneous localizations increased (Table 1). The CNN and FCN networks performed to a similar level with images containing one or two localizations. However, with images containing three or four simultaneous localizations, FCN performed significantly better than CNN (Table 1).

**Figure 8:**
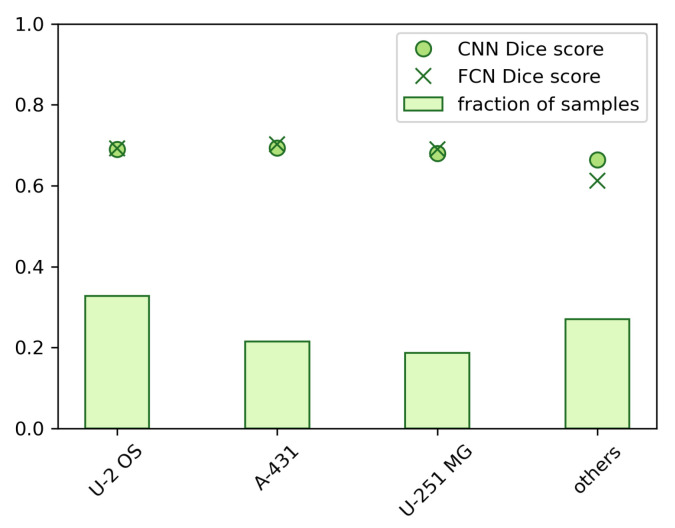
Classification accuracies using the dice coefficient presented separately for samples from the three major cell lines and combined for samples from the other cell lines.

We also studied the effect of different cell lines on the prediction accuracy. As illustrated in Figure 2, the dataset includes samples from 22 different cell lines.Three of the cell lines, ‘U-2 OS’, ‘A-431’ and ‘U-251’ are the origin for majority of the samples (in total 73.5% of all data, 72.9% of test data), whereas the rest <30% are divided between the 19 remaining cell lines. In Figure 8, the prediction accuracy is presented separately for the three dominant cell lines and for a combined class of the other cell lines. We observed a slight drop in the performance for the low-sample cell lines, especially for FCN, suggesting that the heterogeneity due to the differences in cell lines may introduce an additional challenge to the prediction task. See the supplementary repository for Supplementary Figure S1, where the prediction results are presented separately for all 22 cell lines.

**Figure 9:**
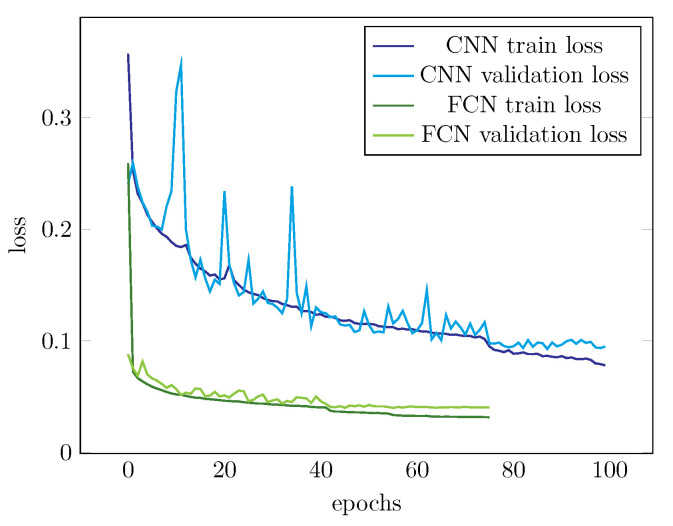
Train and validation losses of both networks.

Finally, we compared the properties of the two network architectures by analyzing their training performance. The decrease of training and validation loss for both network models is presented in Figure 9. It should be noted that the actual values of losses are not comparable since the models output different types of predictions; for CNN the prediction is per cell, whereas for the FCN the prediction is pixelwise, leading to higher number of prediction datapoints and, thus, the effect of individual sample is less dramatic leading to smoother loss curve. We used 20 epoch convergence limit for validation loss decrease as early stopping criterion. This explains why fewer epochs were used for training the FCN model. The major differences between the two networks are the steepness of the decrease of loss in the beginning, and fluctuation scale of validation loss during training. These properties, however, can simply be explained by different scale of loss values, by the difference in the prediction (per cell, fewer datapoints vs. pixelwise, more datapoints) and by the high amount of zero pixels in FCN output. Importantly, both networks reach steady loss towards the end of training, suggesting that training of both networks eventually converged into stable, well performing models.

## 4. Discussion

Here, we studied the applicability and compared the performance of two standard DL-approaches in classification of protein localization from fluorescence microscopy images. We find that both CNN and FCN-type deep neural networks perform well in such a task, and enable AI-based automated phenotyping. The mean of the percentage of correctly classified samples was 0.676 for CNN and 0.696 for FCN. For perspective, the highest $F_{1}$ score in Image Analysis Challenge of Cyto conference 2017 performed on the same data was 0.507, whereas in a large, public image analysis contest [22] the highest reported macro $F_{1}$ scores were around 0.66 for public validation data and 0.59 for private one-off validation dataset, while human expert annotators reach the level of 0.71 (per-class F1, and 0.76 overall F1) [22,31]. We want to emphasize, however, that direct comparison of numerical results reported here and in the contests is not possible due to significant differences in the applied datasets (e.g., number of samples in training/test data, number of classes). Using the unweighted mean of dice coefficients as a metric, our methods score at 0.705 (CNN) and 0.707 (FCN).

From our results it is clear that the localization prediction accuracy is much dependent on in which cellular compartment the protein resides and, especially, whether the localization is heterogenous in relation to cellular structures. The applied dataset is heterogenous in terms of cell lines (Figure 2), which have phenotypic differences that are likely to affect the appearance and relative distances/arrangement of certain cellular locations. For example, the size and shape of the cells, the shape of nuclei, number and appearance of nucleoli, and amount of cytoplasm are known phenotypic differences amongst the cell lines used. Thus, the heterogeneity in the images due to use of multiple cell lines provide a challenge for machine learning algorithms, as also stated in [22]. This may especially influence the results for small groups where cell line heterogeneity is high. On the other hand, training algorithms to deal with the phenotypic heterogeneity is of uttermost importance in producing solutions that have broad applicability.

Not unexpectedly, the classification success was clearly best with images containing only one annotated localization, an observation shared also in [22]. For the proteins residing in several locations at a given time, the performance of the networks may depend on which these locations are, as certain locations reside closer and may even overlap in a 2D image and thus may be more difficult to distinguish from each other. Observing the multilocalization of classes (Figure 7) showed that the classes with most samples (such as cytosol and nucleus) often co-occur with other classes. Comparing the multilocalization classes to the misclassification rates we noted that, although the multilocalizations and misclassifications do not occur at the same frequencies, in many cases the most often misclassified classes are also the ones most often coexisting with other classes. However, based on Figure 4, we can estimate that the misclassifications result from both co-occurrence and vicinity of locations. Even though localization in the nucleus co-occurs frequently with nearly all other classes, and in many cases more frequently than cytosol, the cytosolic compartments are more often misclassified as cytosol than nucleus. However, classification of nucleoli makes an exception. Even though half of samples with nucleoli co-occur with nucleus, nucleoli is separated from nucleus with less than 0.1 misclassification rate. Interestingly, FCN performed significantly better than CNN with images containing three or four localizations (Table 1). This could be explained by how the models handle the samples. CNN provides a single prediction for whole image due to fully connected layers, so the predictions are global in scope of one image. FCN gives local predictions as output, based on field-of-view of each pixel in output. The smaller field-of-view might help find classes that are present in only some of the cells, since global predictions can discard these classes due to their absence in the majority of cells.

Based on our results, we find that the class imbalance in the dataset had a clear effect on the results. The low number of samples in of some of the classes seems to make them more prone to misclassifications—this could be expected as it is well-known that DL-based methods perform particularly well in data-rich settings. For example, actin filaments are often misclassified as nucleus even though they reside on different locations. Many classes are often misclassified as cytosol or nucleus, which are the most common classes in the training data. Class imbalance is a common problem in the field of machine learning, and could be solved with e.g. over- and undersampling or by generating new data of the rare classes. However, with a multi-label problem this is not straightforward, since rare classes often reside with common classes, and oversampling rare classes would also oversample the common classes.

The dataset used here is composed of confocal section images, representing one z-plane each. This may have an effect in the distribution of the signals in the images as it is likely that each image represents the strongest signal plane for the cell/staining in question, creating a possible bias for at least some of the protein localizations. Especially those that are related to protein relative spatial density in z and whose distribution patterns in xy are correlating with variable signal intensities might benefit from data with the full spectrum of protein localization along all z-planes. In the future, a comprehensive set of images representing all z-planes, signal intensities, and localization distributions would allow addressing whether addition of the third dimension to the data would aid classification of proteins of rare classes and with multiple locations.

The task of creating a large-scale annotated dataset is extremely challenging. Based on visual inspection, the annotations in the dataset are likely to contain variation and inaccuracies as missing and/or misclassifed phenotypes. In [31], a gamification and crowd-sourcing-based approach using non-expert online gamers for annotating the images was presented. The performance level of human experts was reported as 0.71 in [31], and it is likely that rare classes and multiple co-occurring localization patterns within an image are a difficult task for the non-expert gamers. In [38], the consistency and variation of protein localization annotations was studied systematically, as the difficulty of the visual recognition task and the variation in localization patterns are likely to lead to errors in annotations. Given the rather early version (HPAv14) used here, it is likely that the annotations have been improved to date. False annotations cause uncertainty both on model training and validation phase.

In [22], the results from a public challenge for protein localization prediction are summarized, representing another crowd-sourcing experiment to gain interest from the machine learning community towards the topic. Such approaches, together with rapidly improving automated classification tools, are very valuable for the community and are advancing the field towards accurate, automated cell characterization and classification.

Even though the performances of the models studied here are similar when comparing total scores, they differ when looking at individual samples. In addition, we observed that the thresholds for CNN were overall much smaller than thresholds for FCN. The predictions of CNN often had lower confidence value, which translates directly to threshold values. Thus, it is essential to select thresholds for each class in a data-driven manner based on the predictions. While the accuracies of CNN and FCN are very similar, a major benefit from using FCN is that it not only predicts which class is present, but also where it locates. Even though the output of FCN is considerably smaller than the original image, we can roughly estimate which cells the protein locates to. When considering the variation between cells in one sample, the knowledge of which cells contain the specific protein in specific compartment can be biologically meaningful. While CNN and FCN were found to perform well, it would be beneficial to create an ensemble of the networks. By combining the class predictions of CNN and predictions with class locations of FCN, the classification accuracy could possibly be further improved.

## Figures and Tables

**Table 1:** Comparison of results based on multilocalization of proteins. Localizations refers to the number of classes present in an image.

Localizations	All Data (%)	Test Data (%)	Test Data (Amount)	Correct % (CNN)	Correct % (FCN)
1	60.6	60.6	2424	92	90
2	33.3	32.8	1313	57	59
3	5.8	6.2	249	25	41
4	0.28	0.35	14	0	29

## Data Availability

Not applicable.

## Supplementary Materials

The following are available at https://www.mdpi.com/2218-273X/11/2/264/s1.

## Abbreviations

The following abbreviations are used in this manuscript:

AI	Artificial intelligence
CNN	Convolutional neural network
DL	Deep learning
ELU	Exponential linear unit
FCN	Fully convolutional neural network
ReLU	Rectified linear unit
SGD	Stochastic gradient descent
