# Supplementary figures and images for: Convolutional Neural Network-Based Artificial Intelligence for Classification of Protein Localization Patterns
Source: Biomolecules. 2021 Feb 11;11(2):264. doi: 10.3390/biom11020264 (PMC7916854; doi:10.3390/biom11020264)

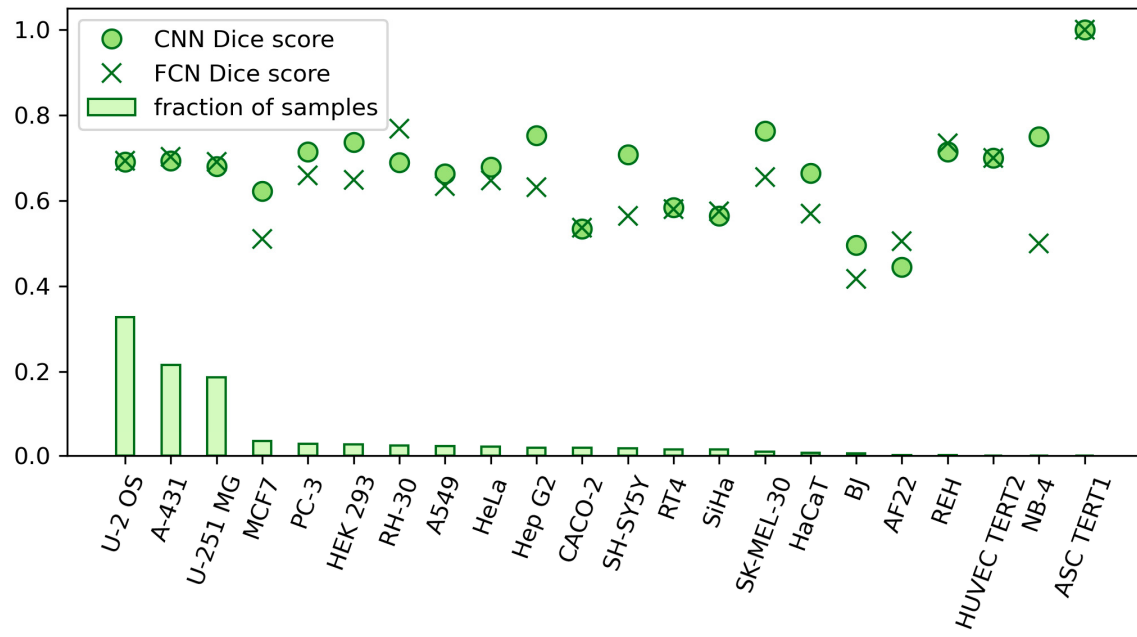

Figure S1

Supplement: Supplementary file 1 [file biomolecules-11-00264-s001.pdf]
